# Effects of Gender and Age on Development of Concurrent Extrapulmonary Tuberculosis in Patients with Pulmonary Tuberculosis: A Population Based Study

**DOI:** 10.1371/journal.pone.0063936

**Published:** 2013-05-22

**Authors:** Chun-Yu Lin, Tun-Chieh Chen, Po-Liang Lu, Chung-Chih Lai, Yi-Hsin Yang, Wei-Ru Lin, Pei-Ming Huang, Yen-Hsu Chen

**Affiliations:** 1 Division of Infectious Diseases, Department of Internal Medicine, Kaohsiung Medical University Hospital, Kaohsiung, Taiwan; 2 Graduate Institute of Medicine, Tropical Medicine Research Center, College of Medicine, Kaohsiung Medical University, Kaohsiung, Taiwan; 3 School of Pharmacy, Kaohsiung Medical University, Kaohsiung, Taiwan; 4 Division of Statistical Analysis, Department of Medical Research, Kaohsiung Medical University Hospital, Kaohsiung, Taiwan; 5 Division of Chest Medicine, Department of Internal Medicine, Kaohsiung Medical University Hospital, Kaohsiung Medical University, Kaohsiung, Taiwan; The Ohio State University, United States of America

## Abstract

Most cases of adult-onset tuberculosis (TB) result from reactivation of a pre-existing *Mycobacterium tuberculosis* infection. *Mycobacterium tuberculosis* usually invades the respiratory tract and most patients develop intrapulmonary TB; however, some patients develop concurrent pulmonary and extra-pulmonary TB. The purpose of the present study was to identify the demographic and clinical factors associated with an increased risk of concurrent extra-pulmonary diseases in patients with pulmonary TB. We compared patients who had isolated pulmonary TB with patients who had concurrent pulmonary and extra-pulmonary TB. We initially analyzed one-million randomly selected subjects from the population-based Taiwan National Health Insurance database. Based on analysis of 5414 pulmonary TB patients in this database, women were more likely than men to have concurrent extra-pulmonary TB (OR: 1.30, *p* = 0.013). A separate analysis of the Kaohsiung Medical University Hospital database, which relied on sputum culture-proven pulmonary TB, indicated that women were more likely than men to have concurrent extra-pulmonary TB (OR: 1.62, *p* = 0.039). There was no significant gender difference in extra-pulmonary TB for patients younger than 45 years in either database. However, for patients 45 years and older, women were more likely than men to have concurrent extra-pulmonary TB (insurance database: 9.0% vs. 6.8%, *p* = 0.016, OR: 1.36; hospital database: 27.3% vs. 16.0%, *p* = 0.008, OR = 1.98). Our results indicate that among patients who have pulmonary TB, older females have an increased risk for concurrent extra-pulmonary TB.

## Introduction

Tuberculosis (TB) is a major threat to public health worldwide and is currently the second leading cause of death from infectious disease, after HIV/AIDS [1]. During the infection process, *Mycobacterium tuberculosis* senses environmental changes and adjusts its physiology to survive in its human host [2]. Only 5% to 10% of *M. tuberculosis–*infected individuals actually develop TB [3], and most adult-onset cases result from reactivation of dormant infections [4], suggesting that interactions between the host and *M. tuberculosis* bacilli clearly affect the development of TB [5].

*M. tuberculosis* usually invades a human host via the respiratory tract, and about 80% of TB patients are diagnosed with pulmonary TB (PTB) [3]. However, some PTB patients present with concurrent extrapulmonary TB (EPTB), a more serious condition. This suggests that the immune systems of PTB patients who develop concurrent EPTB cannot prevent *M. tuberculosis* bacilli from extending beyond the lung parenchyma. Analysis of the epidemiological characteristics of TB patients with different severities of disease may advance our understanding of the causes of EPTB [4].

In the past decade, some cohort studies have reported an increased percentage of EPTB in patients with TB [6], [7]. Several cohort studies investigated the impact of gender on the occurrence of EPTB and found that women were more likely to present their active TB as EPTB [6]–[8]. This gender effect is thought to be related to many factors, such as access to healthcare, socioeconomic factors, and cultural factors. In addition, there is some evidence that sex hormones, genetic factors, and nutritional status play roles in this disparity [9]. Some researchers have suggested that the presence of certain endocrine factors may minimize the severity of TB in females [8], [9], although the underlying factors have not been definitively identified.

Several studies have compared the characteristics of patients with PTB and those with EPTB [10]–[12]. Young age and female gender were found to be risk factors for EPTB in developing countries [11]. The occurrence and type of EPTB was also shown to be dependent on geographical factors suggesting a role for host genetic factors [13]. However, a simple comparison of these two groups may fail to account for the effect of certain local factors (e.g., smoking or chronic lung disease) on the development of disease [13]. No previous study has specifically compared patients with PTB alone with patients who have PTB and concurrent EPTB, and it is not clear what characteristics of the host allow *M. tuberculosis* bacilli to overcome and concurrently infect extrapulmonary sites. It is also not clear if the drug-susceptibility of the pathogen impacts its presentation in human host (isolated PTB versus extension to concurrent EPTB). The purpose of the present study was to identify the demographic and clinical factors associated with increased risk of concurrent extra-pulmonary disease in patients with PTB. The findings from the present study will also be useful for the design of future research on the immune systems of patients with isolated PTB and PTB with concurrent EPTB.

## Materials and Methods

### Design

#### This study used two databases

We initially obtained information from the nationwide Taiwan National Health Insurance (NHI) database and then from a single medical center (the Kaohsiung Medical University Hospital), to investigate the demographic and clinical differences between PTB patients with and without concurrent EPTB. This study design reduced the effect of local factors, because all patients had PTB. This design also enabled a secondary data analysis from two sources to validate our findings.

### Data Sources

#### National health insurance research database

The first database used in this study included one million randomly selected subjects from the 1996–2007 Taiwan National Health Insurance Research Database (NHIRD), which was developed for research purposes. The NHI program was implemented in Taiwan in 1995 and has since provided a comprehensive, unified, and universal health insurance program to all citizens, including adults and children. The NHIRD, which includes data on ambulatory care, hospital inpatient care, dental services, and prescription drugs, is one of the largest and most complete nationwide population-based datasets in Taiwan, and has been used in previous studies [14]. There were no statistically significant differences in age, gender, or average insured payroll-related amount between the sample group and all enrollees.

In Taiwan, PTB diagnoses are based on clinical manifestations (including response to anti-TB treatment), radiological evidence, and laboratory findings. Positive sputum smear results or microbiological cultures are not necessarily required [15]. We enrolled all patients encoded with ICD-9 (International Classification of Diseases, 9th revision) codes 010–018, who received at least two of five first-line anti-TB agents (isoniazid, rifampicin, pyrazinamide, ethambutol, and streptomycin) for more than 60 days within 180 days of diagnosis. Patients with ICD-9 code 011 without any other code between 012 and 018 throughout the anti-TB treatment were considered to have isolated PTB. Those with an ICD-9 code 011 and at least one code between 012 and 018 throughout the anti-TB treatment were classified as having PTB and concurrent EPTB.

#### Hospital database

One disadvantage of the NHI database is that it does not include information of symptoms/signs, underlying diseases personal habits, medications taken, microbiological status of sputum smears (maximal result for individual patients), microbiological status of anti-TB drugs susceptibilities or chest radiographs, making it impossible to perform multivariate analyses on the data. We therefore also used a second database that did contain microbiological data. We retrospectively investigated all patients who were diagnosed with PTB at the Kaohsiung Medical University Hospital (KMUH) between 1 January 2005 and 31 December 2008. KMUH is a 1600-bed tertiary care teaching hospital in Taiwan. We included all patients with positive *M. tuberculosis* complex cultures from respiratory specimens and who were diagnosed as having active PTB. Our study population also included patients who were <15 years old. Culture-negative cases were excluded even if they had positive sputum smears. The Institutional Review Board (IRB) of Kaohsiung Medical University Hospital approved this study, and written informed patient consent was waived by the IRB due to the retrospective nature of the study.

We only included newly diagnosed patients from the second database. Newly diagnosed TB patients (including those with relapse) were defined as patients who were not on active anti-TB therapy at presentation and were not transferred from another healthcare facility with a known diagnosis of active TB. This ensured that all patients included in the second database received thorough clinical investigation of PTB and EPTB at the KMUH. Patients grouped as smokers included both current and ex-smokers. “Alcohol use” was defined as habitual alcohol consumption [13]. Patients were considered to have had steroid exposure if they received steroid treatment consisting of 10 mg or more of prednisolone per day within 14 days of presentation. All medical records were retrospectively reviewed. Each chest radiograph was interpreted by the patient’s own clinician and was independently reviewed by a board-certified radiologist and a pulmonologist. The presence of cavitations was recorded.

The diagnosis of concurrent EPTB was made according to the guidelines of the American Thoracic Society and Centers for Disease Control and Prevention [16]. In brief, patients were diagnosed with EPTB if *M. tuberculosis* complex was isolated from an extra-pulmonary site or if there were pathologic findings of granulomatous inflammation combined with positive acid-fast bacilli staining in tissue biopsy specimens [13]. PTB patients without concurrent EPTB were diagnosed as having isolated PTB. Furthermore, TB is a notifiable disease in Taiwan. Thus, we re-evaluated each patient’s diagnosis after the completion of anti-TB therapy until 31 December 2010 (24 months after the last patient was diagnosed with PTB), based on data generated from the registration system of the Taiwan Centers for Disease Control. This measure provided a chance to categorize our patients correctly during treatment. The final diagnoses were used for assignment of patients to the isolated PTB or the PTB with concurrent EPTB groups.

Pleural TB is considered a form of EPTB, so PTB patients with concurrent pleural TB were also categorized in the concurrent EPTB group. Although pleural involvement may result from direct extension of *M. tuberculosis* complex from lung parenchyma [17], it is an indication that the immune system cannot prevent *M. tuberculosis* bacilli from extending beyond the lung parenchyma, as with non-pleural cases of EPTB [8], [18]. Patients who presented with radiographic findings characteristic of miliary TB were categorized as having EPTB. Besides, based on the prevalence of HIV in Taiwan (0.1%) [19], [20], HIV serostatus, when unknown or undocumented, was conservatively considered to be negative for the purposes of statistical analysis [17].

### Statistical Analysis

All analyses were conducted using SPSS software version 14.0 (SPSS Inc., Chicago, IL). The independent two samples *t-*test was used to compare continuous variables. The associations of EPTB with other categorical variables were tested with the Chi-square test or Fisher’s exact test. The strengths of associations of EPTB with other categorical variables were presented by odds ratios (ORs) with 95% confidence intervals (CIs). Variables with *p*-values less than 0.10 in the univariate analysis were included in the multivariate logistic regression model. All tests were two-tailed, and *p*-values less than 0.05 were considered statistically significant.

## Results

### National Health Insurance Research Database and Hospital Database

**Figure 1:**
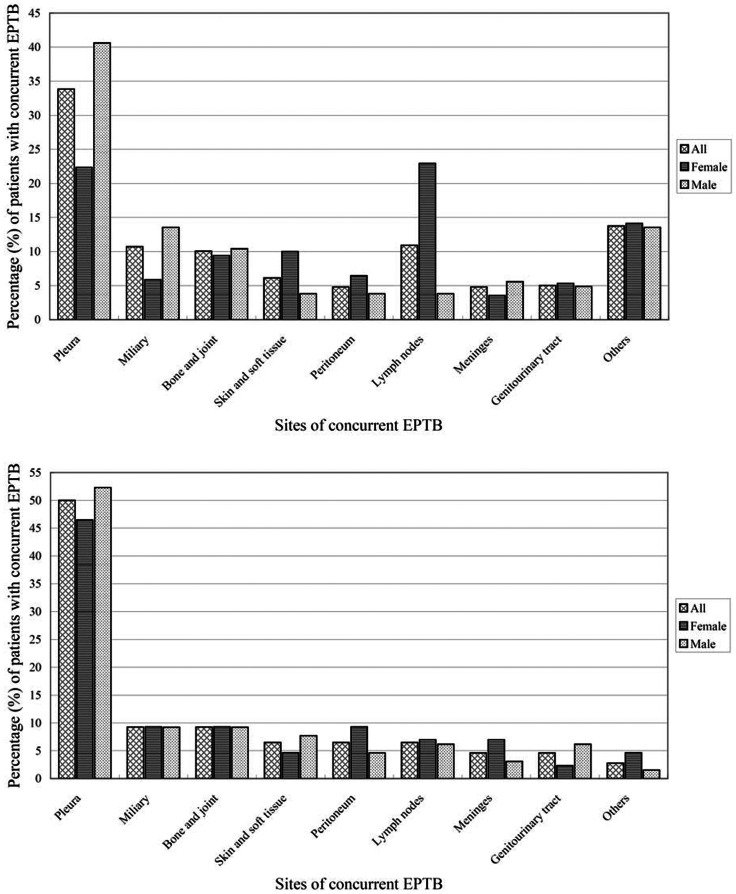
Sites of concurrent EPTB involved among patients with PTB, from the NHI database (upper panel) and the KMUH database (lower panel).

Analysis of one-million randomly selected patients from the NHIRD indicated that 5,414 patients (3581 males [66.1%] and 1833 females [33.9%]) were diagnosed with PTB and received more than 60 days of treatment with at least two anti-TB agents. There were totally 4980 patients (92.0%) with isolated PTB and 434 patients (8.0%) who had PTB with concurrent EPTB. The most common EPTB site was the pleural space (155 patients), followed by lymphatic (50 patients) and miliary (49 patients), as shown in Table 1 and Figure 1A. A flow chart depicting the selection of patients with PTB from the NHIRD is presented as Figure S1.

**Table 1:** Demographic characteristics of PTB patients, from the NHI database (*n* = 5414) and KMUH database (*n* = 547).

	NHI database	KMUH database	*p*
	(*n* = 5414)	(*n* = 547)	
Age (years), mean ± standard deviation	58.3±19.5	62.8±18.4	<0.001
Age range (years)	0–99	2–96	
Sex, female, *n* (%)	1833 (33.9)	167 (30.5)	0.175
Concurrent EPTB patients, *n* (%)	434 (8.0)	110 (20.1)	<0.001
**Involved extra-pulmonary sites, ** ***n*** ** (% of PTB patients with concurrent EPTB)**			
Pleural	155 (35.7)	54 (49.1)	
Lymphatic	50 (11.5)	9 (8.2)	
Miliary	49 (11.3)	10 (9.1)	
Osteoarticular bone and/or joint	46 (10.6)	10 (9.1)	
Skin and soft tissue	28 (6.5)	7 (6.4)	
Genitourinary	23 (5.3)	5 (4.5)	
Peritoneal	22 (5.1)	7 (6.4)	
Meningeal	22 (5.1)	6 (5.5)	
Others	63 (14.5)	4 (3.6)	

During the study period, 547 patients of the KMUH were newly diagnosed with PTB. HIV serologic test results were obtained from 65 patients and six patients were positive. As shown in Table 1, the mean age of KMUH patients was 62.8±18.4 years. Three hundred and eighty patients (69.5%) were male, and 167 patients (30.5%) were female. These numbers are comparable to those of the general population data in Taiwan [21]. Among the 547 culture-confirmed TB cases, five patients (0.9%) were infected by multidrug-resistant *M. tuberculosis*, and 81 patients were infected by *M. tuberculosis* which was resistant to at least one first-line anti-TB drug. Patients in the KMUH database were significantly older than those in NHI database, possibly because that KMUH was a referral medical centre catering to a larger number of older patients. The percentage of PTB patients having concurrent EPTB was also significantly higher in KMUH database, possibly because patients who visited the medical centre had an expectation of receiving thorough/comprehensive clinical investigation of concurrent PTB and EPTB. However, there was no significant difference in gender distribution between the two databases (Table 1).

### Association of Gender with PTB+EPTB

In the NHIRD, a total of 171 females (9.3%) had PTB with EPTB, and 263 males (7.3%) had PTB with EPTB (female-to-male OR: 1.30; 95% CI: 1.06–1.60; *p* = 0.013). Among patients younger than 45 years, we found no significant association between gender and the presence of PTB+EPTB. In contrast, for patients who were 45 years old and older, significantly more females than males had PTB with concurrent EPTB (107 of 1185 cases [9.0%] *vs.* 192 of 2829 cases [6.8%], *p* = 0.016). A similar result was observed in the hospital database, where significantly more females than males had PTB with EPTB were observed in those 45 years and older (27.3% vs. 16.0%, *p* = 0.008). (Table 2).

**Table 2:** The occurrence of concurrent EPTB in patients with PTB by age and gender groups, from NHI and KMUH database.

		Age <45 years			Age ≥45 years		
		Male	Female	P-value	Male	Female	P-value
NHI database	PTB+EPTB	71 (9.4%)	64 (9.9%)	0.854	192 (6.8%)	107 (9.0%)	0.016
*n* = 5414	isolated PTB	681 (90.6%)	584 (90.1%)		2,637 (93.2%)	1,078 (91.0%)	
KMUH database	PTB+EPTB	15 (27.8%)	8 (20.5%)	0.577	52 (16.0%)	35 (27.3%)	0.008
*n* = 547	isolated PTB	39 (72.2%)	31 (79.5%)		274 (84.0%)	93 (72.7%)	

**Figure 2:**
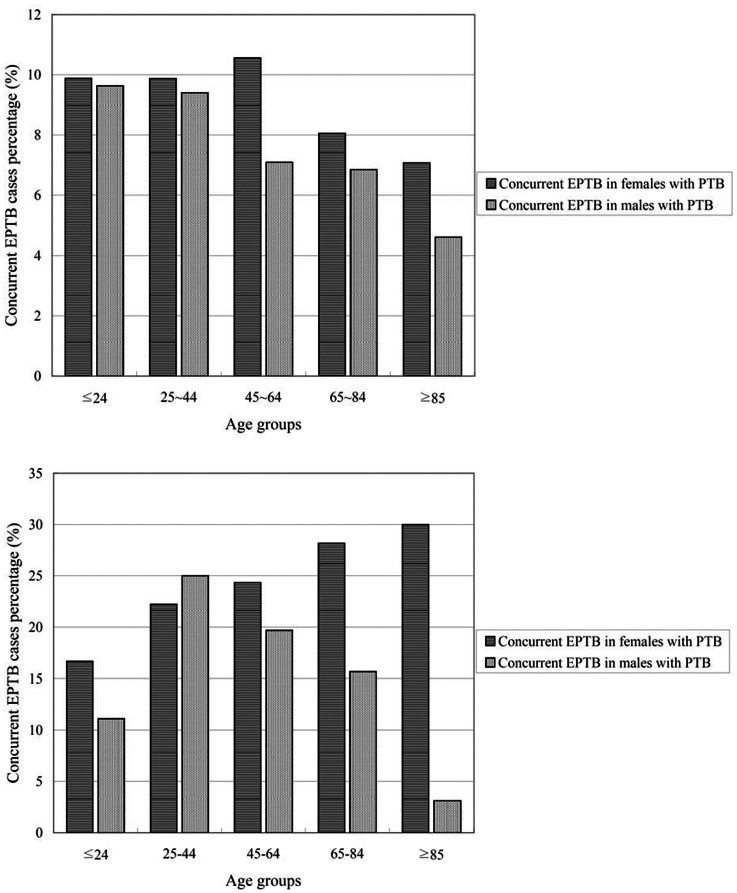
Percentage of PTB patients who had concurrent EPTB, by age groups, from the NHI database (upper panel) and the KMUH database (lower panel).

We further stratified the patients in five age groups to observe the association between gender and PTB+EPTB. We found no significant association between gender and the presence of PTB+EPTB in patients aged ≤24 and 25– 44 years old. However, women were more likely than men to have concurrent extra-pulmonary TB among patients aged between 45 to 64 years old, 65 to 84 years old and ≥85 years old (Figure 2A and 2B).

### Univariate and Multivariate Analyses for the Hospital Database

Among the 547 PTB patients, 437 patients (79.9%) had isolated PTB and 110 patients (20.1%) had PTB with concurrent EPTB. The most common EPTB site was the pleural space (54 patients), followed by miliary and osteoarticular bone and/or joint involvement (10 patients each), as shown in Table 1 and Figure 1B. The diagnosis of EPTB was confirmed by culture in 49 patients and by consistent pathologic findings of tissue biopsy specimens in 61 patients. Of the 49 EPTB cultures, all drug susceptibility test results agreed with those for respiratory specimens from the same patient.

Univariate analysis of the demographic factors and clinical manifestations of these patients indicated that women had higher likelihoods of having PTB with EPTB. However, variables traditionally thought to be correlated with a heavy burden of pulmonary *M. tuberculosis* bacilli (i.e., positive sputum smears and cavitary lesions on chest radiographs [22]) were not significantly different in patients with isolated PTB and those with PTB and concurrent EPTB (Table 3).

**Table 3:** Univariate analysis of factors associated with concurrent EPTB in PTB patients, from the KMUH database (*n* = 547).

	No. (%) of patients			
Variable	With EPTB	Without EPTB	Odds ratio	*p*
	(*n* = 110)	(*n* = 437)	(95% CI)	
**Demographic characteristics**				
Sex, female	43 (39.1)	124 (28.4)	1.62 (1.05–2.51)	0.039
Age ≥45 years	87 (79.1)	367 (84.0)	0.72 (0.43–1.22)	0.281
**Symptoms/signs**				
Cough ≥3 weeks	48 (43.6)	233 (53.3)	0.68 (0.44–1.03)	0.087
Sputum production	43 (39.1)	200 (45.8)	0.76 (0.50–1.17)	0.249
Dyspnea	19 (17.3)	51 (11.7)	1.58 (0.89–2.81)	0.158
Fever >38.0°C	55 (50.0)	200 (45.8)	1.18 (0.78–1.80)	0.491
Weight loss	13 (11.8)	69 (15.8)	0.71 (0.38–1.35)	0.372
**Underlying diseases**				
Respiratory disorders	11 (10.0)	69 (15.8)	0.59 (0.28–1.20)	0.157
Previous tuberculosis history	6 (5.5)	30 (6.9)	0.78 (0.32–1.93)	0.750
Malignancy	18 (16.4)	80 (18.3)	0.87 (0.50–1.53)	0.737
Diabetes mellitus	30 (27.3)	121 (27.7)	0.98 (0.61–1.56)	0.999
HIV co-infection	4 (3.6)	2 (0.5)	8.20 (1.48–45.45)	0.019
Connective tissue disease	3 (2.7)	4 (0.9)	3.04 (0.67–13.70)	0.300
Others	33 (30.0)	117 (26.8)	1.17 (0.74–1.86)	0.577
**Personal habits**				
Smokers	39 (35.5)	183 (41.9)	0.76 (0.48–1.20)	0.264
Alcohol use (habitual alcohol consumption)	21 (19.1)	114 (26.1)	0.67 (0.40–1.13)	0.162
**Medications exposure**				
Steroids	4 (3.6)	20 (4.6)	0.79 (0.26–2.35)	0.799
**Microbiological status of sputum smears (maximal result for individual patients)**				
Smear positive results	61 (55.5)	260 (59.5)	0.85 (0.56–1.29)	0.508
Acid-fast stain 4+	19 (17.3)	100 (22.9)	0.70 (0.39–1.25)	0.252
Acid-fast stain 3+	8 (7.3)	37 (8.5)	0.85 (0.35–1.97)	0.831
Acid-fast stain 2+	16 (14.5)	66 (15.1)	0.96 (0.51–1.79)	0.998
Acid-fast stain 1+	18 (16.4)	57 (13.0)	1.30 (0.70–2.40)	0.453
**Microbiological status of anti-TB drug susceptibilities**				
Multidrug (rifampin and isoniazid) resistant	1 (0.9)	4 (0.9)	0.99 (0.11–9.01)	0.999
Resistant to at least one first-line drug resistant	20 (18.2)	61 (14.0)	1.37 (0.79–2.39)	0.335
**Chest radiographs**				
Cavitation	24 (21.8)	95 (21.7)	1.01 (0.61–1.67)	0.999

CI, confidence interval.

In addition, the percentage of *M. tuberculosis* strains resistant to anti-TB agents was not significantly different between the isolated PTB group and the PTB and concurrent EPTB group. Furthermore, we entered all variables that had *p*-values less than 0.10 in univariate analyses (Table 3) into the multivariate logistic regression model (Table 4). Our results indicated that gender, HIV co-infection, and cough ≥3 weeks were the three independent factors influencing EPTB: women were more likely than men to have concurrent EPTB (adjusted OR = 1.75, p = 0.013); PTB patients with HIV co-infection were more likely to have concurrent EPTB (adjusted OR = 11.83, p = 0.005); and those with cough ≥3 weeks were less likely to have concurrent EPTB (adjusted OR = 0.63, p = 0.034).

**Table 4:** Multivariate analysis of factors associated with concurrent EPTB in patients with PTB from the KMUH database.

Variable	Adjusted odds ratio (95% CI)	*p*
**Demographic characteristics**		
Gender, female	1.75 (1.13–2.73)	0.013
**Symptoms/signs**		
Cough ≥3 weeks	0.63 (0.41–0.96)	0.034
**Underlying diseases**		
HIV co-infection	11.83 (2.09–66.99)	0.005

CI, confidence interval.

## Discussion

In this study, we showed that among PTB patients, females were more likely to have concurrent EPTB than males, and this gender inequality was mostly observed in patients ≥45 years old compared to those <45 years old. This was seen in both databases.

The initial site of *M. tuberculosis* infection is usually the respiratory tract, and non-pulmonary initial infection is very rare. Since numerous factors affect the susceptibility of patients to clinically active TB, a comparison of the demographic and clinical characteristics of different patient groups may help to identify mechanisms underlying host susceptibility and defence [4]. Previous studies have noted the emergence of EPTB as an important form of active TB [10], [23], and that the percentage of EPTB among TB patients has increased in the past decade [6], [7]. The pathogenesis of EPTB may differ from that of PTB [5], [17], and many studies have compared patients with isolated EPTB and patients with isolated PTB. However, no studies have specifically compared the demographic and clinical characteristics of PTB patients with and without concurrent EPTB. There is evidence that the different lesions of TB patients with multisystem involvement are caused by the same strain of *M. tuberculosis* [24], [25]. These data suggest an inability of the immune systems of patients with concurrent PTB and EPTB to confine *M. tuberculosis* bacilli to the lung parenchyma. A comparison of patients who have isolated PTB with patients who have concurrent PTB and EPTB may help to identify factors that increase the risk for more severe TB.

Previous studies reported gender differences in the development of TB [26]–[28]. The prevalence of *M. tuberculosis* infection is similar in males and females until the age of 15 years, after which it is greater in males [29]. Analysis of sex-specific TB incidence rates in San Francisco also showed a male/female ratio of 2.1 [26]. However, EPTB presents differently than PTB. Studies in the United States [6], [28], Germany [8], Denmark [30], and India [7] all reported that females were more likely than males to have EPTB. Several factors were thought to be related to this inequality, such as endocrine factors [8], smoking, and TB exposure [11]. Smoking is a risk factor for TB and PTB [31], and smokers are less likely to have isolated EPTB than non-smokers [31]. According to the “Taiwan Tobacco Control Annual Report 2010” [32], the prevalence of smoking was 9-fold higher in males than females in Taiwan. Thus, smoking may be a confounding factor in a simple comparison of isolated PTB and isolated EPTB. In other words, it is difficult to conclude that gender-related differences (such as endocrine factors) or smoking were responsible for men being more likely than women to have PTB. In our study, we only analyzed patients with PTB in order to decrease the impact of local pulmonary confounding factors, such as smoking and chronic lung disease [9], [13]. This study design allowed us to better assess the effect of gender and host immune status on the development of EPTB. A remarkable advantage of using the KMUH database was that it included detailed drug susceptibility test data, making it possible to clarify the possible effect of drug resistance on the degree of disease involvement.

Our findings suggest that the immune systems of older women are less able to contain bacilli locally in the lung parenchyma. The average age for menopause is 51 years, and the levels of sex hormones begin to drop several years before this (perimenopause) [33]. The proportion of PTB patients with concurrent EPTB was significantly greater in females older than 45 years, suggesting that hormonal factors may play a role in the greater susceptibility of older women to EPTB.

The absence of a cough ≥3 weeks was independently more likely in patients with PTB and concurrent EPTB compared to patients with isolated PTB, suggesting that the disease in these patients developed at extrapulmonary sites, such as bones and joints, and they present symptoms or signs at an extrapulmonary site or present systemic manifestations. Moreover, not surprisingly, PTB patients co-infected with HIV were independently more likely to have concurrent EPTB. TB patients co-infected with HIV have significantly disrupted cell-mediated immunity [34] and such patients are more likely to have EPTB [35].

The percentage of patients with PTB and concurrent EPTB among all PTB patients has been reported by a number of groups as 5.8% (North Carolina, United States) [12], 6.1% (Madagascar) [10], 16.5% (Nepal) [11], and 19.6% (Minnesota, United States) [23]. Although a significantly greater percentage of patients in the KMUH cohort had concurrent PTB and EPTB, the percentages of both our cohorts (8.0% in the NHI database and 20.1% in the KMUH database) were still comparable to these previous studies. As already noted earlier, the percentage of TB patients who develop EPTB has increased over the past decade [6], [7]. This may be due to a slower decrease in EPTB than PTB, improvements in diagnostic methods, or improved public health management of PTB [6]. Since 2003, the regulations of the Centers for Disease Control of Taiwan have required all clinicians in Taiwan to consider the possibility of PTB in patients who are diagnosed with EPTB. Our two databases were from different time periods (NHI: 1996 to 2007; KMUH: 2005 to 2008), so this policy change may partially explain the difference in the percentage of patients with concurrent EPTB. Regardless, our analysis of the two databases indicated the same impact of gender and age on the development of EPTB in patients with PTB.

Although the pleura was the most commonly involved extra-pulmonary site of concurrent EPTB in both databases, the lymph nodes represented another important site involved in concurrent *M. tuberculosis* among women in the NHIRD. Our data agreed with previous studies investigating EPTB (regardless of whether it was isolated or concurrent with PTB), which also found that pleura and lymph nodes were the leading sites affected by *M. tuberculosis* bacilli [6]–[8], [10], [11], [27].

It is important to note that previous studies compared PTB patients with EPTB patients, while our study compared patients with PTB group and patients who have PTB with concurrent extra-pulmonary disease. Our study has some limitations. First, although the NHIRD is population-based, not all of the TB patients were diagnosed based on bacteriological results. Instead, patients were included based on clinical diagnoses made by individual physicians and the administration of anti-TB treatment for at least 60 days. Patients who died within 60 days after diagnosis would therefore have been missed. Second, in the KMUH cohort, despite the use of comprehensive clinical investigations, it was not possible to definitively exclude extra-pulmonary diseases in all patients. For example, mycobacterial cultures of urine are not routine in clinical practice. In the present study, we verified all diagnoses after completion of anti-TB therapy according to the registration system of the Taiwan Centers for Disease Control. It is also important to note that since it is a retrospective study based on the information from an existing database, we could not remedy the situation whereby a large percentage of patients did not have their HIV status checked in the past. Third, we did not record the menopausal status of patients, and this limits our interpretation of the role of hormonal factors on the development of EPTB.

In conclusion, we sought to identify factors associated with the development of EPTB by comparison of patients with isolated PTB and patients with PTB and concurrent EPTB. This study design aimed to avoid possible pulmonary confounding factors. For patients with PTB, females were more likely than males to have concurrent EPTB, especially among patients ≥45 years old. There was no significant role of “*M. tuberculosis* resistance” and “traditional PTB bacterial burden markers” in the development of concurrent EPTB, which suggested that host factors rather than microorganism factors played a role in disease progression. Our findings suggested that female PTB patients of post-reproductive age are more susceptible to concurrent EPTB than male patients of the same age group. The role of endocrine factors in the pathogenesis of TB requires further investigation. Regardless, our results indicate that older women with PTB should be more carefully investigated for the presence of concurrent EPTB and that prolonged or more intensive treatment should be considered for these patients.

## Supporting Information

Figure S1 **Flow chart of selection of patients with PTB from NHIRD.**
(TIF)
