# Correction: Effects of Gender and Age on Development of Concurrent Extrapulmonary Tuberculosis in Patients with Pulmonary Tuberculosis: A Population Based Study

**DOI:** 10.1371/annotation/c4731438-54d3-48b4-ad22-1b8f0d2d57ef

**Published:** 2013-07-09

**Authors:** Chun-Yu Lin, Tun-Chieh Chen, Po-Liang Lu, Chung-Chih Lai, Yi-Hsin Yang, Wei-Ru Lin, Pei-Ming Huang, Yen-Hsu Chen

The number of the grant from Kaohsiung Medical University Hospital is incorrect. The correct grant number is: KMUH100-0M08. 

