# Supplementary figures and images for: Effects of Gender and Age on Development of Concurrent Extrapulmonary Tuberculosis in Patients with Pulmonary Tuberculosis: A Population Based Study
Source: PLoS One. 2013 May 22;8(5):e63936. doi: 10.1371/journal.pone.0063936 (PMC3661599; doi:10.1371/journal.pone.0063936)

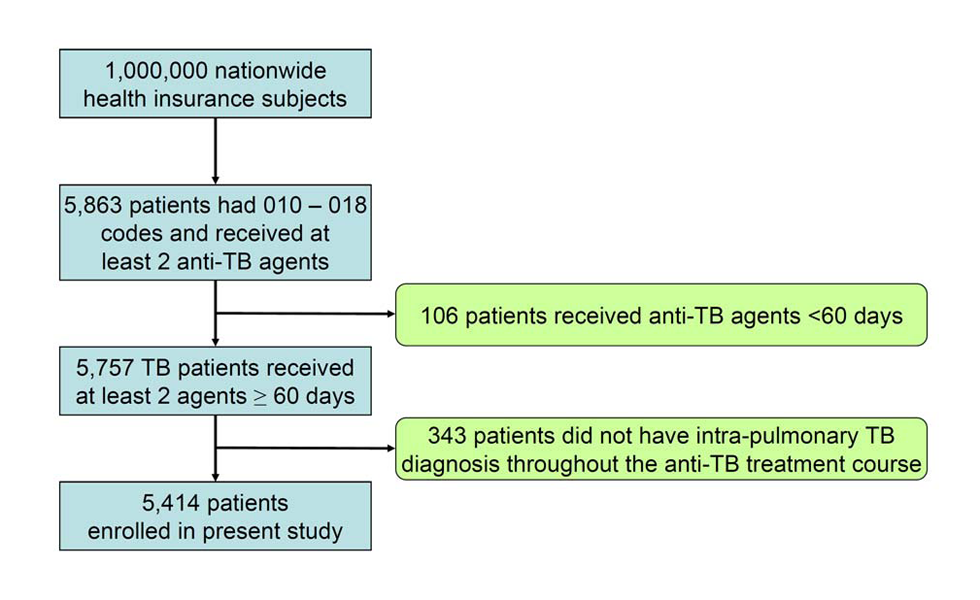

Supplement: Figure S1 — Flow chart of selection of patients with PTB from NHIRD. (TIF) [file pone.0063936.s001.tif]
